# Selective targeting of melanoma using *N*-(2-diethylaminoethyl) 4-[^18^F]fluoroethoxy benzamide (4-[^18^F]FEBZA): a novel PET imaging probe

**DOI:** 10.1186/s13550-017-0311-2

**Published:** 2017-08-08

**Authors:** Pradeep K. Garg, Rachid Nazih, Yanjun Wu, Vladimir P. Grinevich, Sudha Garg

**Affiliations:** 1 Wake Forest University Health Sciences, Winston Salem, NC USA; 2 Center for Molecular Imaging and Therapy, Biomedical Research Foundation, 1505 Kings Highway, Shreveport, LA 71133 USA; 3 Current Address: Asinex Corporation, 10 N. Chestnut Street, St 104, Winston Salem, NC 27101 USA

**Keywords:** Melanoma, 4-[^18^F]FEBZA, Radiochemical synthesis, Radiofluorination, Binding affinity, Cell binding, Biodistribution, MicroPET imaging

## Abstract

**Background:**

The purpose of this study was to develop a positron emission tomography (PET) imaging probe that is easy to synthesize and selectively targets melanoma in vivo. Herein, we report the synthesis and preclinical evaluation of *N*-(2-diethylaminoethyl) 4-[^18^F]Fluoroethoxy benzamide (4-[^18^F]FEBZA).

A one-step synthesis was developed to prepare 4-[^18^F]FEBZA in high radiochemical yields and specific activity. The binding affinity, the in vitro binding, and internalization studies were performed using B16F1 melanoma cell line. The biodistribution studies were performed in C57BL/6 normal mice, C57BL/6 mice bearing B16F1 melanoma tumor xenografts, and nu/nu athymic mice bearing HT-29 human adenocarcinoma tumor and C-32 amelanotic melanoma tumor xenografts. MicroPET studies were performed in mice bearing B16F1 and HT-29 tumor xenografts.

**Results:**

4-[^18^F]FEBZA was prepared in 53 ± 14% radiochemical yields and a specific activity of 8.7 ± 1.1 Ci/μmol. The overall synthesis time for 4-[^18^F]FEBZA was 54 ± 7 min. The in vitro binding to B16F1 cells was 60.03 ± 0.48% after 1 h incubation at 37 °C. The in vivo biodistribution studies show a rapid and high uptake of F-18 in B16F1 tumor with 8.66 ± 1.02%IA/g in this tumor at 1 h. In contrast, the uptake at 1 h in HT-29 colorectal adenocarcinoma and C-32 amelanotic melanoma tumors was significantly lower with 3.68 ± 0.47%IA/g and 3.91 ± 0.23%IA/g in HT-29 and C-32 tumors, respectively. On microPET images, the melanoma tumor was clearly visible by 10 min post-injection and the intensity in the tumor continued to increase with time. In contrast, the HT-29 tumor was not visible on the microPET scans.

**Conclusions:**

A rapid and facile synthesis of 4-[^18^F]FEBZA is developed. This method offers a reliable production of 4-[^18^F]FEBZA in high radiochemical yields and specific activity. A high binding affinity to melanoma cells and high uptake in tumor was noted. The microPET scan clearly delineates the melanoma tumor by 10 min post-injection. The results from these preclinical studies support the potential of 4-[^18^F]FEBZA as an effective probe to image melanoma.

## Background

An estimated 87,000 new cases of melanoma of the skin will be diagnosed in the USA in 2017, and approximately 9730 individuals are anticipated to die of melanoma this year [1]. Among many factors, lacking monthly self-inspection of the skin by patients, routine visits to the dermatologist combined with lack of effective diagnostic tools training for the accurate detection of melanoma at an early stage, malignant melanoma remains a significant health problem and remains as the sixth most prevalent type of cancer [1]. While melanoma accounts for a small percentage of all skin cancer cases, 75% of deaths from skin cancer are associated with melanoma. Among many factors, late diagnosis and poor localization of metastatic lesions in patients with melanoma results in high mortality from this cancer [2]. If diagnosed at stage 1 of this disease, the overall 10-year survival for melanoma patients is ~95%. By contrast, if this disease is diagnosed at stage IV, the overall survival drops to 10–15% [2]. Therefore, early diagnosis and accurate assessment of metastatic lesions is crucial for appropriate treatment planning, improved outcome, and disease-free survival [3].

Positron Emission Tomography (PET) is a powerful tool to non-invasively study organ function and related disorders such as tissue perfusion and metabolism in vivo [4]. PET imaging with F-18 fluorodeoxy glucose (FDG) has shown high sensitivity and specificity in the diagnosis and staging of various cancers [5] primarily due to its significantly higher uptake in malignant cells with elevated metabolic activity [6]. While quite effective in many types of cancers, FDG-PET has shown a low mean sensitivity of 17.3% for detecting small lymph node metastases in patients with early-stage melanoma cancer [6–8], thus offering limited value in the initial staging of AJCC (American Joint Commission on Cancer) stage I, or stage II melanoma [6, 9, 10]. In another study, FDG-PET failed to detect melanoma in all 14 patients showing a positive sentinel lymph node biopsy for cancer [11]. Additionally, the high uptake of FDG in brain tissues further limits its utility in detecting brain metastases [12]. Therefore, developing a PET probe with high specificity and selectivity for melanoma and a low uptake in normal tissues has been the goal of many investigators [13–15].

To selectively target melanoma, the role of F-18- and Cu-64 labeled α–melanocyte-stimulating hormone analog to image melanoma by targeting melanocortin-1 receptors was evaluated [16–19] but showed limited success. In addition, potential of various fluorinated iodoquinoxaline carboxamide was also explored as imaging and therapeutic probe to target melanoma [20]. Additionally, the efforts were made to develop radioiodinated benzamides as potential melanoma-targeting agents [21–24], partially inspired from the incidental finding of a high uptake of iodo-benzamides in uveal tissues of pigmented experimental animals [24]. With the wider acceptance of PET as a clinical diagnostic tool for accurate diagnosis as well as encouraging findings on the ability of radioiodobenzamide to target melanoma, there has been a significant impetus to develop PET probes which incorporate benzamide moiety [17, 20, 25–30]. One such compound, ^18^F-DAFBA, which was initially reported by our group [25] and subsequently explored by other investigators, showed promising results [26]. Nonetheless, the synthesis of DAFBA requires ~3 h and includes two or more high-performance liquid chromatography (HPLC) purifications [25]. To overcome that hurdle, we subsequently developed ^11^C-MBZA with a shorter and easier synthesis [31]. Preclinical evaluations of this ^11^C-probe showed superior in vitro and in vivo properties compared to that for ^18^F-DAFBA. While ^11^C-MBZA has many desirable features and properties, it incorporates a short half-life radionuclide (*T*_1/2_ = 20 min). Therefore, we focused our efforts towards developing an F-18 benzamide with a short and facile radiochemical synthesis akin to ^11^C-MBZA while bearing similar or better biological properties. Herein, we report the synthesis, radiochemistry, and preclinical evaluation of 4-[^18^F]FEBZA as a PET probe to selectively and non-invasively target melanoma.

## Methods

All chemicals were purchased from Sigma (Sigma-Aldrich, Milwaukee, WI) and were used without further purification. C-18 plus Sep-Pak cartridges were obtained from the Waters Corporation (Waters Corporation, Milford, MA). Flash column chromatography was carried out over silica gel (60 Å, Mallinckrodt Baker). HPLC was performed in the isocratic mode using an HPLC system (Varian Corp, Palo Alto, CA) equipped with an LC pump, a variable wavelength UV/VIS detector set at 254 nm, and an in-line radioisotope detector (Bioscan Inc., Washington, DC). The HPLC column for the purification of 4-[^18^F]FEBZA was a C-18 reverse-phase, 10 mm × 250 mm, 5-μ Luna column (Phenomenex, Torrence, CA), and elution was performed at a flow rate of 2 mL/min using a solution of methanol:0.1 M ammonium acetate (50:50) containing trimethylamine (40 μL/100 mL). The quality of the purified product was checked using a C-18 column (4.6 mm × 250 mm, 5 μ), eluted at a flow rate of 1 mL/min using methanol:0.1 M ammonium acetate (50:50) containing trimethylamine (40 μL/100 mL). ^18^F-Fluoride was produced by the ^18^O(p,n)^18^F nuclear reaction using O-18 enriched water (95% enrichment) on a GE PET-Trace 800 series cyclotron (GE Healthcare, Milwaukee, WI). Kryptofix solution was prepared by dissolving 120 mg of K_2.2.2._ and 720 mg of K_2_CO_3_ in 720 μL of water followed by the addition of 12 mL of acetonitrile. The accell plus QMA-cartridge was activated by successively flushing it with 5 mL of 1 N sodium bicarbonate solution and 10 mL of sterile water, followed by air flushing. The C-18 plus cartridge was activated by washing with 5 mL of ethanol followed by 10 mL of sterile water.

B16F1 melanoma cells, HT-29 human colorectal adenocarcinoma cells, and C-32 amelanotic melanoma cells were purchased from the American Type Culture Collection (ATCC, Rockville, MD). B16F1 murine melanotic melanoma cells were maintained in Dulbecco’s modified Eagles medium supplemented with 10% fetal calf-serum, 2 mM L-glutamine, and 1 mM pyruvate. HT-29 and C-32 cells were maintained in RPMI supplemented with 10% fetal calf serum and 2 mM L-glutamine. C57BL/6 and athymic nu/nu mice were purchased from Charles River Laboratories (Charles River Laboratories, Boston, MA). All animal studies were performed using an IACUC-approved protocol, and the work was performed under the guidelines established by Wake Forest University. The cells were grown to confluency, harvested, washed with phosphate-buffered saline and then were suspended in a 1:1 mixture of phosphate-buffered saline and Matrigel (BD Life Sciences, San Jose, CA) at a concentration of 5 × 10^6^ cells/mL. For subcutaneous tumor xenografts, 6 mice were inoculated/6 mice were inoculated in the right flank with 100 μL of a suspension of B16F1 cells, and female athymic nu/nu mice were injected in the right flank with 100 μL of HT-29 cells or C-32 cells.

### Preparation of 1-fluoro 2-tolunesulphonyloxyethane

To a solution of 2-fluoroethanol (480 mg, 7.52 mmol) in dry dichloromethane (100 mL) were successively added p-toluenesulfonyl chloride (2.16 g, 11.28 mmol), triethylamine (1.6 mL, 11.28 mmol), and 4-dimethylaminopyridine (108 mg, 0.88 mmol). After stirring the reaction mixture for 8 h at room temperature, the contents were washed with 1 M HCl (50 mL) and water (2 × 50 mL). The organic layer was separated, dried over anhydrous magnesium sulfate, filtered and evaporated to dryness. The residue was purified by silica gel flash column chromatography (10% ethyl acetate in hexane).

### Preparation of *N*-(2-diethylaminoethyl) 4-hydroxybenzamide (4-HBZA; *1*)

This intermediate was synthesized as described previously [31]. Briefly, a mixture of thionyl chloride (30 mL) and 4-acetoxy benzoic acid (7.4 g, 43 mmol) in dimethylformamide (5 mL) was refluxed for 4 h. After removal of thionyl chloride on a rotary evaporator, triethylamine (6 mL, 43 mmol) and *N,N*-diethylethylenediamine (5 g, 43 mmol) were added slowly at 0 °C, and the contents were stirred overnight at room temperature. After the usual work-up of the reaction, 50 mL of sodium methoxide in methanol (0.5 M) was added followed by stirring the reaction mixture for 24 h at room temperature. After evaporating the solvent, the residue was dissolved in 200 mL of dichloromethane and was washed successively with water (2 × 50 mL), 1 M sodium bicarbonate (2 × 20 mL), and water (2 × 100 mL). The resulting crude product was purified using silica gel flash column chromatography (5% methanol in dichloromethane).

### Preparation of *N*-(2-diethylaminoethyl) 4-(2-tolunesulphonyloxyethoxy) benzamide (*3*)

A suspension of NaH (120 mg, 5 mmol) in tetrahydrofuran (THF) (50 mL) was cooled to 0 °C, and a solution of 4-HBZA (*1*) (590 mg, 2.5 mmol) in 20 mL of THF was added. After stirring the reaction mixture for 10 min, a solution of 1,2-di-toluenesulphonyloxyethane (1.85 g, 5 mmol) in 10 mL of THF was added, and the reaction mixture was refluxed overnight. The contents were extracted in 100 mL of dichloromethane, and the organic layer was washed with 1 M HCl followed by 2 × 50 mL of water. Dichloromethane was removed, and the residue was purified using flash column chromatography (5% methanol in CH_2_Cl_2_). The molecular formula was as follows: C_22_H_30_N_2_O_5_S. ^1^H NMR (CDCl3, δ). ^1^H NMR (CDCl_3_, δ): 1.33 (t, 6H, 2 × N–(CH_2_–C*H*_3_)_2_), 2.36 (s, 3H, C*H*_3_–Ar–SO_2_), 3.17 (q, 4H, 2 × N–(C*H*_2_–CH_3_)_2_), 3.33 (t, 2H, −C*H*_2_–N–(CH_2_–CH_3_)_2_), 3.82 (m, 2H, −CONH–C*H*_2_–), 4.12 (m, 2H, Ar–O–C*H*_2_–CH_2_–), 4.36 (m, 2H, Ar–O–CH_2_–C*H*_2_–), 7.17 (m, 2H, Ar–*H*–C3 and C5–), 7.27 (m, 2H, Ar–*H*–C2 and C6). ESI-MS m/z [MH]+ calcd 434, found 435.19.

### Preparation of *N*-(2-diethylaminoethyl) 4-fluoroethoxybenzamide (4-FEBZA; *2*)

A suspension of NaH (120 mg, 5 mmol) in tetrahydrofuran (50 mL) was cooled to 0 °C, and a solution of 4-HBZA (590 mg, 2.5 mmol) in tetrahydrofuran (20 mL) was added. After stirring the reaction mixture for 10 min, a solution of 1-fluoro-2-tosyloxyethane (1.09 g, 5 mmol) in 10 mL of tetrahydrofuran was added, and the reaction mixture was refluxed overnight. After adding 100 mL of dichloromethane, the contents were washed with 1 M HCl followed with 2 × 50 mL of water. After drying the organic layer over anhydrous magnesium sulfate, the solvent was removed, and the residue was purified using flash column chromatography (5% methanol in CH_2_Cl_2_) to afford the desired product. The molecular formula was as follows: C_15_H_23_N_2_O_2_F. ^1^H NMR (CDCl_3_, δ): 1.07 (t, 6H, 2 × −CH_2_–C*H*_3_), 2.61 (q, 4H, 2 × −*CH*_2_ CH_3_), 2.70 (t, 2H, −C*H*_2_–N–(CH_2_–CH_3_)_2_), 3.50 (q, 2H, −CONH–CH_2_–), 4.25 (m, 2H, F–CH_2_–C*H*_2_), 4.78 (m 2H F–C*H*_2_–CH_2_), 6.95 (m, 2H, Ar–*H*–C3 and C5–), 7.78 (m, 2H, Ar–*H*–C2 and C6). ESI-MS m/z [MH]+ calcd 282; found 283.29.

### Radiochemical synthesis of *N*-(2-diethylaminoethyl) 4-[^18^F]fluoroethoxybenzamide (4-[^18^F]FEBZA; *4*)

After irradiation, the ^18^O–H_2_O bolus containing 450–800 mCi of [^18^F]fluoride was transferred from the cyclotron target to a pre-activated QMA cartridge in the hot cell. The trapped [^18^F]fluoride was eluted from the cartridge with 1.5 mL of kryptofix (K_2.2.2._) solution prepared as described previously [32]. The remaining water from this mixture was removed azeotropically using acetonitrile. The dried [^18^F]fluoride thus obtained was reacted with a freshly prepared solution of 1–5 mg of precursor *3* in 200 μL of anhydrous DMSO or acetonitrile at 115 °C for 5–20 min. Subsequently, the contents were diluted with 1 mL of water and loaded on to a reverse phase semi-preparatory HPLC column. The product was eluted from the column into a flask containing 70 mL of water. Subsequently, the contents were transferred and trapped onto a C-18 Sep-Pak, and the cartridge was washed with 10 mL of water to remove solvent residue. The desired product was eluted from the Sep-Pak with 1 mL of ethanol followed by 9 mL of saline and collection in a sterile product vial.

#### In vitro binding assay

The binding affinity of 4-[^18^F]FEBZA was measured using the homologous completion assay in 12-well plates containing a uniform number of fixed B16F1 cells/well. After adding 0.1 nmol of 4-[^18^F]FEBZA/well as the radioligand, unlabeled 4-FEBZA (2) was added to the quadruplicate sets of wells in concentrations ranging from 10^−11^ to 10^−4^ M and the plates were incubated for an hour at 37 °C under 5% CO_2_. At the end of the incubation, the media were removed, and the cells were washed with 500 μL of ice-cold media. The cells were lysed using 1 mL of phosphate-buffered saline containing 10% Triton X-100, and the lysates were collected in culture tubes to measure the radioactivity contents using a γ-counter. We performed a non-linear regression analysis of the decay-corrected CPM values to calculate the inhibition constant value (IC_50_), the concentration of competitor required to inhibit 50% of radiotracer binding, utilizing the GraphPad Prism software program (GraphPad Software, Inc., CA). The binding curve was generated by fitting the data simultaneously to one-site and two-site competitive binding models, and the *F* test provided a preferred model for the mean IC_50_ values (confidence interval 95%).

The in vitro cell binding was assessed by growing B16F1 cells in culture flasks to 80–90% confluence, trypsinizing, washing in PBS (pH 7.4), aliquoting into 12-well plates and allowing adherence to the wells overnight. Initially, we studied the influence of the incubation time and temperature on the binding to B16F1 melanoma cells. For this study, 12-well plates containing ~2.5 × 10^5^ cells/well were incubated at 0, 22, and 37 °C with 4-[^18^F]FEBZA in quadruplicate sets for 15, 30, 60, 120, and 180 min. After the incubation, the media were removed, and each well was washed with fresh ice-cold media (2 × 2 mL). The adherent cells were removed by lysing cells in 500 μL of PBS containing 10% Triton X-100 (Sigma-Aldrich, St. Louis, MO). The combined washes and lysate were analyzed for radioactivity content using a γ-counter.

Next, we evaluated the binding characteristics of 4-[^18^F]FEBZA to B16F1 cells through internalization experiments [33, 34]. The cells were treated with a low pH buffer to sequester membrane-bound radioactivity (acid wash-releasable) from the internalized (acid wash-resistant) fraction [34]. For this assay, 4-[^18^F]FEBZA was added to 12-well plates with each well containing 4 × 10^5^ B16F1cells/well (in quadruplicate), and the plates were incubated at 37 °C for 15, 30, 60, 120, and 180 min. After the incubation, the media were removed, each well was washed with ice-cold PBS (2 × 1 mL), and the washes were collected in the tubes. Subsequently, each well was gently treated with 1 mL of an ice-cold mildly acidic medium (sodium acetate buffer, pH 4.5) to remove surface-bound radioactivity, and the rinses were collected in tubes (acidic wash). The adherent cells were then lysed using 500 μL of 10% Triton X-100 in PBS, and the lysate was transferred into tubes. The total radioactivity in each of the lysate, acidic wash, and the PBS wash was measured using a γ-counter.

#### Tissue distribution studies

The biodistribution studies were performed in mice bearing B16F1 (1 h *n* = 5; 2 h *n* = 6), C-32 (1 h *n* = 3; 2 h *n* = 3), and HT-29 (1 h *n* = 3; 2 h *n* = 3) tumor xenografts. The tumor-bearing mice were injected with ~40 μCi of 4-[^18^F]FEBZA. A separate group of normal C57BL/6 mice with no tumor implants (1 h *n* = 3; 2 h *n* = 3) was also injected with ~40 μCi of 4-[^18^F]FEBZA to study the distribution characteristics of 4-[^18^F]FEBZA in the absence of tumors. At 1 h and 2 h post-injection, the mice were euthanized, and the tissues of interest were removed, rinsed with PBS, weighed, and counted for radioactivity contents using a γ-counter.

#### MicroPET imaging studies

In vivo imaging of 4-[^18^F]FEBZA in mice bearing B16F1 (*n* = 2) and HT-29 (*n* = 1) tumor was performed using a P4 microPET™ scanner (Concorde Microsystems, Inc., Knoxville, TN). After administering ~100 μCi of 4-[^18^F]FEBZA via tail-vein injection, whole body PET scans were acquired in list mode. The images were reconstructed using filtered back projection with attenuation correction. Regions Of Interest (ROIs) were drawn on select organs using the summed image from the entire scan acquisition session, and the data were analyzed using ASIPro software (Siemens Preclinical Solutions, Knoxville, TN). The time activity curves (TACs) were generated from the list mode data reconstructed into multiple 5-min frames.

## Results

**Fig. 1:**
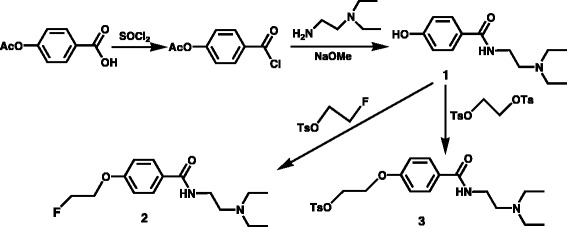
Preparation of the title compound and various other intermediates is shown. Compound (*1*) was prepared following previously published method and is shown above. The synthesis of non-radioactive title compound (*2*) was prepared as the reference standard and was accomplished by reacting compound (*1*) with 1-fluoro 2-toluenesulphonyloxyethane. Compound (*3*) was prepared as precursor for the radiochemical synthesis. The preparation of compound (*3*) was accomplished by reacting compound (*1*) with commercially available ditosylethane

The synthetic procedure to prepare various intermediates and the non-radioactive final product is shown in Fig. 1. The compound 1-fluoro 2-toluenesuIfonyloxyethane was obtained as a colorless oil in 88% yields. The intermediate 4-HBZA (*1*) was prepared in 28% yields as a light-yellow-colored oil following a previously published procedure. The reference standard for the title compound *N*-(2-diethylaminoethyl) 4-fluorothoxybenzamide (*2*) was prepared in 40% yields as an amorphous white compound. The tosylate-precursor (*3*) was prepared as an amorphous white compound in 32% chemical yields.

**Fig. 2:**
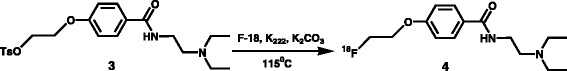
The radiochemical synthesis of 4-[^18^F]FEBZA (*4*) was accomplished using a single-step method and is shown above. The precursor (*3*) was reacted with F-18 fluoride in kryptofix/K_2_CO_3_ and the crude reaction mixture was subsequently purified using HPLC

The radiochemical synthesis of 4-[^18^F]FEBZA (*4*) was accomplished through a simple one-step fluoro-for-tosyl exchange reaction and is shown in Fig. 2. The total time required for the synthesis, HPLC purification, and reformulation of the final product in buffer required less than 1 h. On a semi-prep column, the final product was eluted at ~16–17 min; on an analytical-HPLC column, it eluted at ~8–9 min. The radiochemical yields ranged from 11 to 20% (16 ± 4%) using 1 mg of precursor. The radiochemical yields increased to 53 ± 14% with doubling the precursor amount. No significant change in the radiochemical yields was noted using 5 mg of precursor. The radiochemical purity of 4-[^18^F]FEBZA was >99%, and the specific activity was 8.7 ± 1.1 Ci/μmol. The specific activity of 4-[^18^F]FEBZA was significantly higher than the specific activity reported for ^18^F-FPBZA (30–40 GBq/μMol) and comparable to specific activity reported for MEL050, ^18^F-DAFBA and ^11^C-MBZA [25, 28, 35]. The overall synthesis time for the 4-[^18^F]FEBZA including the HPLC purification and reformulation was 54 ± 7 min.

**Fig. 3:**
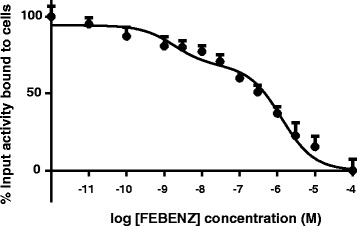
Results from homologs competition of 4-[^18^F]FEBZA for the varying concentration of FEBZA binding to B16F1 cells. The B16F1 cells were incubated with non-radioactive FEBZA (10^−11^ M–10^−4^ M) at room temp for 1 h followed by adding a fixed amount of 4-[^18^F]FEBZA to each well. Each data point (mean ± SD; *n* = 4 wells) represents the percent of added 4-[^18^F]FEBZA bound to the cells in presence of varying concentration of non-radioactive FEBZA

The binding affinity of 4-[^18^F]FEBZA was assessed using a homologous competitive binding assay. The results from this assay are shown in Fig. 3. In this assay, two distinct binding sites were noted for the 4-[^18^F]FEBZA, with IC_50_ values of 1.8 nM and 1.3 μM. A nanomolar binding is an attractive property of this compound, perhaps indicating a high targeting ability of this compound to melanoma.

**Fig. 4:**
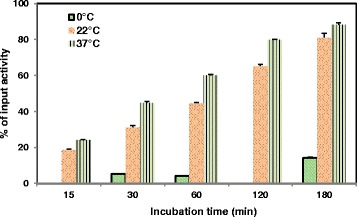
The B16F1 cells (~2.5 × 10^5^ cells/well) were incubated with 4-[^18^F]FEBZA for 15, 30, 60, 120, and 180 min at 0, 22, and 37 °C. Significantly higher binding was noted at 22 and 37 °C as compared to that at 0 °C. The data is expressed as percent of total radioactivity added to each well and the values are the mean ± SD (*n* = 4 wells). Data for 0 °C was determined only at 30, 60, and 180 min

The impact of incubation time and temperature on total binding of 4-[^18^F]FEBZA to melanoma cell was assessed using 2.5 × 10^5^ cells/well using 12-well plates. The total binding to B16F1 cells at 37, 22, and 0 °C was 60.03 ± 0.48%, 44.45 ± 0.61%, and 4.17 ± 0.30%, respectively, and is shown in Fig. 4.

Results from the biodistribution studies performed in C57BL/6 mice bearing B16F1 and athymic nu/nu mice bearing HT-29 and C-32 tumor xenografts at 1 and 2 h are presented in Table 1.

**Table 1:** Biodistribution of 4-[^18^F]FEBZA in mice bearing tumor xenografts

	%IA/g tissue (mean ± SD)					
Organ	B16F1 tumor		HT-29 tumor		C-32 tumor	
	1 h	2 h	1 h	2 h	1 h	2 h
	(*n* = 6)	(*n* = 5)	(*n* = 3)	(*n* = 3)	(*n* = 3)	(*n* = 3)
Liver	3.25 ± 0.34	2.28 ± 0.49	4.84 ± 0.95	2.50 ± 0.29	3.11 ± 0.49	1.99 ± 0.12
Spleen	2.24 ± 0.36	1.58 ± 0.18	2.11 ± 0.25	1.51 ± 0.12	1.96 ± 0.38	1.22 ± 0.08
Lung	2.12 ± 0.17	1.49 ± 0.49	2.19 ± 0.33	1.63 ± 0.16	1.94 ± 0.67	1.33 ± 0.35
Heart	2.82 ± 0.25	2.12 ± 0.58	2.52 ± 0.12	2.19 ± 0.37	2.52 ± 0.15	1.75 ± 0.35
Kidneys	3.10 ± 0.28	2.20 ± 0.38	2.91 ± 0.24	2.08 ± 0.29	2.85 ± 0.37	1.84 ± 0.12
Bone	3.78 ± 0.45	5.45 ± 0.98	3.83 ± 0.39	5.71 ± 1.11	3.47 ± 0.78	5.09 ± 0.72
Sm. Intestine	2.49 ± 0.25	1.60 ± 0.31	1.98 ± 0.62	1.42 ± 0.10	2.23 ± 0.21	1.33 ± 0.06
Pancreas	2.48 ± 0.35	1.73 ± 0.14	2.41 ± 0.26	1.56 ± 0.18	2.49 ± 0.42	1.40 ± 0.10
Muscles	1.51 ± 0.21	1.14 ± 0.21	1.61 ± 0.28	1.40 ± 0.24	2.35 ± 1.06	1.05 ± 0.14
Brain	2.55 ± 0.61	2.25 ± 0.50	1.68 ± 0.18	1.52 ± 0.28	1.87 ± 0.21	1.36 ± 0.17
Adrenals	1.10 ± 0.36	0.88 ± 0.06	1.07 ± 0.08	0.91 ± 0.19	1.26 ± 0.39	0.77 ± 0.18
Blood	3.06 ± 0.52	2.51 ± 0.93	2.80 ± 0.26	2.29 ± 0.23	3.22 ± 0.64	2.40 ± 0.30
Tumor	8.66 ± 1.02	7.47 ± 1.34	3.68 ± 0.47	3.07 ± 0.36	3.91 ± 0.23	2.53 ± 0.19

## Discussion

Towards an ongoing effort to develop a PET imaging probe to target melanoma with high specificity and selectivity, we recently reported the synthesis and preliminary preclinical evaluations of a novel PET imaging probe ^11^C-MBZA. Despite its attractive in vitro binding and in vivo biodistribution characteristics, the short half-life of C-11 radionuclide (*t*_1/2_ = 20 min) remained a small set-back for the wide use of this very promising probe. Our next goal was to develop an imaging probe incorporating a facile and rapid radiochemical synthesis along with biological traits similar ^11^C-MBZA. To that end, we have now developed an F-18-labeled fluoroethoxy analog, i.e., 4-[^18^F]FEBZA, and herein, we present those findings.

The radiosynthesis procedure for 4-[^18^F]FEBZA is quite simple and straight forward. This procedure provides the desired compound in high radiochemical yields and purity. The synthesis time and the radiochemical yields for the 4-[^18^F]FEBZA are quite comparable to those reported for many of the other melanoma-targeting agents reported earlier such as ^18^F-DAFBA [25], F-18-labeled iodoquinoxaline carboxamide [20], ^18^F-FPBZA [28], MEL050, and other F-18-labeled fluoronicotinamides [27]. Additionally, the synthesis of 4-[^18^F]FEBZA is quite adaptable to automation using many of the commercially available synthesis modules.

4-[^18^F]FEBZA showed a rapid and high binding to B16F1 melanoma cells at 37 and 22 °C. In contrast, it showed insignificant binding to B16F1 cells at 0 °C; perhaps indicating the involvement of an active uptake pathway towards its binding to melanoma cells. In addition to temperature dependency, 4-[^18^F]FEBZA also showed a time-dependent increase in binding. A threefold higher binding was noted when the cells were incubated for 60 min as compared to a 15 min incubation. While the cell binding continued to further increase with time, the increase was modest beyond 120 min incubation. The increase in binding for cells incubated at 22 °C was slightly lower than that at 37 °C. For comparison, the overall binding of ^18^F-FPBZA to B16F0 melanoma cells was 4.12 ± 0.20% after 2 h incubation at 37 °C, a 20-fold lower binding despite a higher number of cells (1 × 10^6^ cells) used in the assay [28].

**Fig. 5:**
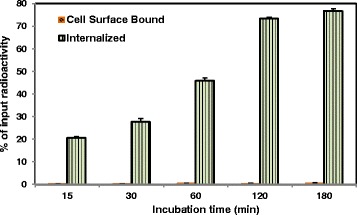
Internalization of 4-[^18^F]FEBZA into B16F1 cells (~5 × 10^5^ cells/well) at 37 °C. The data represents the percent of total cell-associated radioactivity and is the mean ± SD (*n* = 4). Majority of the cell-associated radioactivity is internalized and the cell surface bound fraction amount is negligible (<1%) at all time points and the remainder of the radioactivity was present in the media. The internalized fraction increased with time and reached plateau by about 120 min

In addition to rapid and high binding of 4-[^18^F]FEBZA to B16F1 melanoma cells, the majority of the cell-associated radioactivity was internalized and the results from this assay are shown in Fig. 5. A combination of attributes such as the high IC_50_ value and strong internalization could be beneficial and further allude to the potential of 4-[^18^F]FEBZA as an effective melanoma targeting probe.

A two- to threefold higher uptake of radioactivity was noted in B16F1 melanoma tumors as compared to that in the HT-29 or the amelanotic C-32 tumors. The ^18^F-FEBZA uptake in B16F1 tumor is quite similar to tumor uptake reported for various other fluorinated benzamides. For example, the F-18 uptake in B16F1 tumor at 1 h was 8.66 ± 1.02%IA/g, 8.4 ± 1.5%IA/g, and 8.31 ± 1.00%IA/g for the ^18^F-FEBZA, MEL050, and ^18^F-FPBZA, respectively [25, 26, 28, 35]. Similarly, the uptake of these three compounds in non-melanoma tumor was low.

The biodistribution studies show a low uptake of 4-[^18^F]FEBZA in normal tissue and the uptake levels continued to decrease further with time, leading to significantly reduced radioactivity levels in normal tissues. For example, the uptake in the liver and the lungs from B16F1 tumor-bearing mice at 1 h was 3.25 ± 0.34%IA/g and 2.12 ± 0.17%IA/g, respectively, and these levels decreased to 2.28 ± 0.49%IA/g and 1.49 ± 0.49%IAD/g, respectively, by 2 h. The radioactivity contents in the small intestine of mice bearing B16F1, HT-29, and C-32 tumors was 2.48 ± 0.35%IA/g, 1.98 ± 0.62%IA/g, and 2.23 ± 0.21%IA/g, respectively, at 1 h. Uptake in intestine seems high but radioactivity levels in intestine was comparable to uptake levels seen in other organs. The intestinal uptake for ^18^F-DAFBA, MEL050, and ^18^F-FPBZA at 1 h in mice bearing B16F melanoma tumor was 3.04 ± 0.24%IA/g, 2.1 ± 0.3%IA/g, and 2.45 ± 0.38%IA/g, respectively.

Additionally, the uptake levels in the liver, lungs, intestine, and several other organs were comparable between the three types of tumor-bearing mice used in this study. For a radiotracer to qualify as good imaging agent, it should exhibit low uptake in normal tissues along with a rapid and high uptake in the tumor. A high lipophilicity of the compound has been identified as one of the many factors responsible for higher uptake in normal tissues. Therefore, a radiotracer with lower lipophilicity would be desirable to minimize accumulation in the normal tissues, but could alongside, has the potential to reduce its uptake in the tumor. Therefore, a good balance between lipophilicity and functional groups within the molecular moiety is a key when designing biologically active imaging agents.

**Fig. 6:**
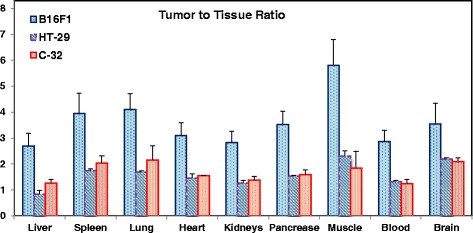
Tumor-to-normal tissue ratio at 1 h is presented for the mice bearing B16F1, C-32, and HT-29 tumors. The tumor-to-tissue ratio for the amelanotic C-32 and colorectal adenocarcinoma HT-29 group of mice were quite similar and these ratios were appreciably lower than that for the mice from B16F1 group. A twofold lower tumor-to-muscle ratio was noted for the C-32 and HT-29 as compared to that from B16F1 group

The radioactivity levels decreased in normal tissues with time. The uptake in B16F1 tumor was rapid, high and the tumor uptake levels plateaued by 1 h. A combination of these two factors resulted in favorable tumor to tissue ratios for the melanoma group as compared to those for the HT-29 tumor group. The tumor-to-tissue ratio at 1 h post-injection for various groups is presented in Fig. 6. The tumor-to-muscle ratio at 2 h for the B16F1, C-32, and HT-29 mice groups was 6.64 ± 1.11, 2.43 ± 0.14, and 2.21 ± 0.17, respectively. The tumor-to-tissue ratio for the 4-[^18^F]FEBZA is quite comparable to those reported for ^18^F-DAFBA and other ^11^C-labeled and ^18^F-labeled benzamides [20, 25–27].

A low uptake of radioactivity was noted in most normal tissues except for the bones. A high uptake in the bones is generally an indirect measure of in vivo defluorination [25, 36, 37], thus indicating metabolic dehalogenation of 4-[^18^F]FEBZA. Although high bone uptake has been reported for MEL050 (0.58 ± 0.05%IA/g at 2 h) and ^18^F-FPBZA (3.21 ± 0.16%IA/g at 2 h), high bone uptake observed for the ^18^F-FEBZA is somewhat unexpected since the structurally close analog ^18^F-DAFBA showed low bone uptake (0.20 ± 0.06%IA/g at 2 h) [26]. Placement of fluorine group on the alkyl chain of 4-[^18^F]FEBZA may be a likely contributor towards enhanced defluorination. The covalently bound fluorine to aryl group in ^18^F-DAFBA and MEL050 may have provided extra stability to Aryl–F bond towards catabolic defluorination in vivo. To further investigate whether the enhanced defluorination of ^18^F-FEBZA and subsequent accumulation of ^18^F-fluoride in the bone was tumor mediated phenomenon, we repeated the biodistribution study in C57BL/6 mice with no implanted tumor. The bone uptake at 1 h in mice with no tumor, and the mice with B16F1, HT-29, and C-32 tumors was 3.23 ± 0.24%IA/g, 3.78 ± 0.45%IA/g, 3.83 ± 0.39%IA/g, and 3.47 ± 0.78%IA/g, respectively (*p* > 0.1). In addition, the uptake of 4-[^18^F]FEBZA in various organs was similar in mice with no tumors and the tumor bearing mice. For example, at 1 h %IA/g in the liver, lungs, muscle, blood, and brain was 3.25 ± 0.34, 2.12 ± 0.17, 1.51 ± 0.21, 3.06 ± 0.52, and 2.55 ± 0.61, respectively, in mice bearing B16F1 tumor (Table 1) and 4.11 ± 0.63, 2.52 ± 0.47, 1.10 ± 0.13, 2.81 ± 0.31, and 2.46 ± 0.41, respectively, in mice without the implanted tumor. The differences in tissue uptake for the two groups are statistically insignificant (*p* > 0.1). Similarity in tissue distribution data and bone uptake levels for the two groups of mice indicate that the defluorination might be resulting from weaker C–F alkyl bond and perhaps not due to tumor mediated catabolic defluorination of the parent molecule.

The ability of a melanoma imaging agent to cross the blood brain barrier is considered another important feature of probes to target metastatic lesions to the brain. The tissue distribution studies showed a modest uptake of radioactivity in the mouse brain (2.55 ± 0.61%IA/g at 1 h) demonstrating the ability of this compound to cross the blood-brain barrier. In comparison, the uptake of MEL050 in mouse brain as determined from microPET imaging studies was 1.38 ± 0.40%IA/g at 60–70 min [38]. More importantly, the levels in the brain were significantly lower than those in the tumor. The tumor-to-brain tissue ratios for the B16F1 and HT-29 groups were 3.55 ± 0.80 and 2.19 ± 0.04, respectively, at 1 h, and 3.45 ± 0.81 and 2.04 ± 0.19, respectively, at 2 h. A high tumor-to-brain ratio (Fig. 6) further supports the ability of 4-[^18^F]FEBZA to delineate metastatic lesions present in the brain.

**Fig. 7:**
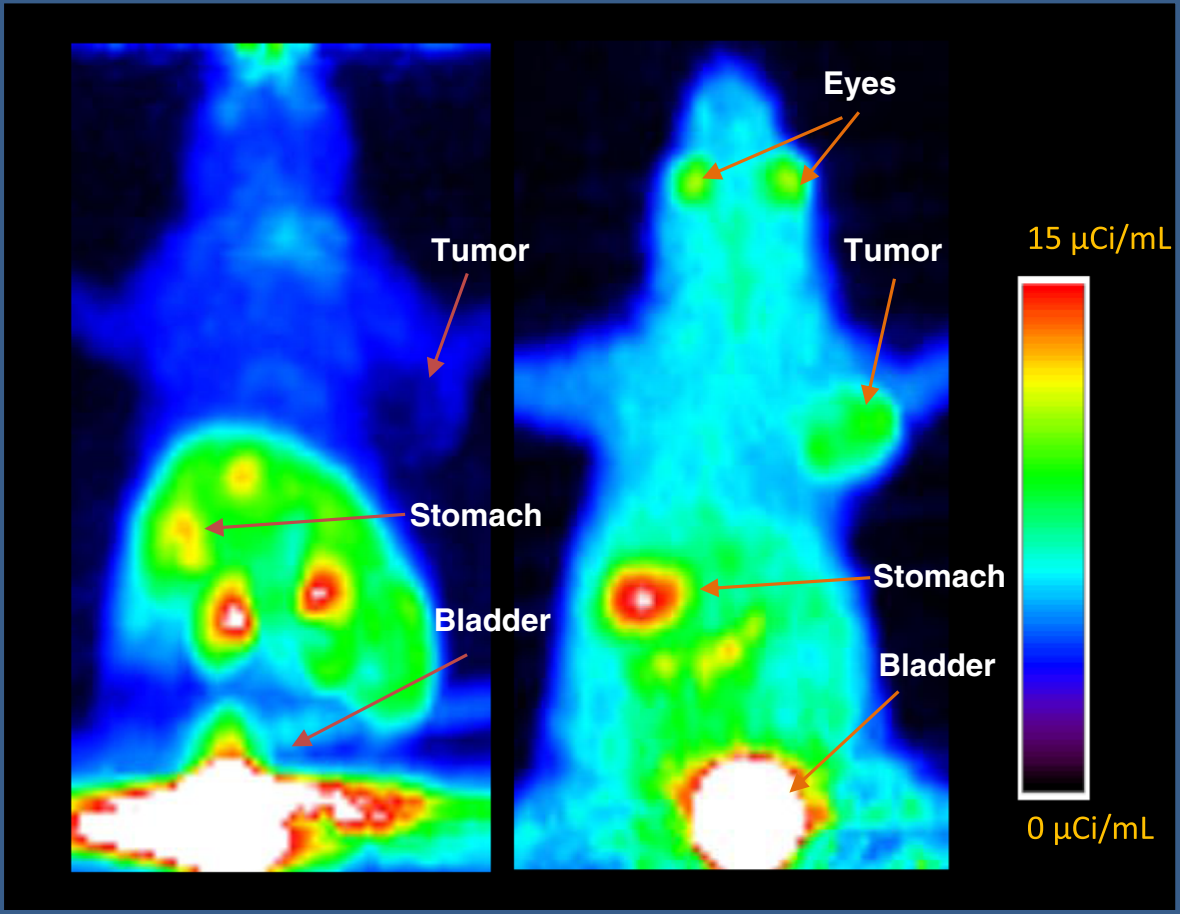
Maximum intensity projection (MIP) image from MicroPET scans of mice bearing B16F1 melanoma (*right panel*) and HT-29 human colorectal (*left panel*) tumor xenograft. An intense uptake of radioactivity is seen in B16F1 tumor. In contrast, HT 29 tumor shows a significantly low uptake and it is quite difficult to visualize this tumor on microPET images (*left panel*). Eyes show a distinct uptake pattern in B16F1 tumor bearing mice (*right panel*). Additionally, the uptake of radiotracer in skin of black mice (C57/BL6) was evident from darker background (*right panel*) in comparison to lack of such uptake in the skin for HT-29 tumor bearing athymic mice (*left panel*)

Based on these encouraging results, we further explored the imaging characteristics of 4-[^18^F]FEBZA through microPET studies in mice bearing B16F1 and HT-29 tumor xenografts. The maximum intensity projection (MIP) images and the axial view from microPET images acquired at 30 min for mice bearing B16F1 and HT-29 tumor xenografts is shown in Fig. 7. In order to provide appropriate comparison between the two independently acquired microPET imaging studies, each image set was normalized to injected dose levels (Fig. 7). The normalized MIP projection over coronal display is that MIP images minimize the frame selection bias and enables direct comparison between the two subjects. As anticipated, the MIP images show distinct uptake of 4-[^18^F]FEBZA in the eyes and tumor of mouse bearing B16F1 tumor xenograft. In addition, the black skin coat of the C57BL/6 mouse is visible due to binding of this radiotracer to skin containing melanin (Fig. 7, right panel). In contrast, the skin of athymic mice does not contain melanin and thus show lack of F-18 accumulation in the skin (Fig. 7, left panel). Most other melanoma-targeting agents display similar characteristic uptake in the eyes and the skin of C57BL/6 mice, a unique feature of this family of compounds [27, 28, 39]. On microPET images, B16F1 tumor was clearly visible on the summed image. In contrast, the HT-29 tumor was not discernable.

**Fig. 8:**
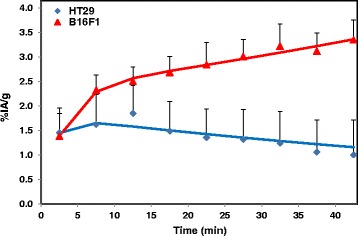
Time activity curves showing tumor uptake in mice bearing B16F1 and HT-29 tumors. The curves were generated from microPET images. Each data point represents the percent of injected Activity per gram (%IA/g) of B16F1 tumor (*red*) and HT-29 (*Blue*) at various time points after injecting 4-[^18^F]FEBZA. After an initial accumulation in both the tumor, the uptake levels in melanoma tumor increased with time (*red*), whereas the levels in HT-29 tumor decreased and remained low (*blue*)

To further understand the uptake characteristics, time activity curves were generated from the ROIs drawn over the organs visible on microPET images. The time activity curves for 4-[^18^F]FEBZA uptake in B16F1 melanoma tumor and HT-29 (colorectal carcinoma tumor) are shown in Fig. 8. After an initial uptake, the radioactivity levels in B16F1 tumor increased with time reaching over 3.5%IA/g tumor by 47 min, whereas, the levels in HT-29 tumor decreased significantly with time; resulting in threefold higher uptake in B16F1 tumor (Fig. 8). It is noteworthy that the tumor uptake values reported as %IA/g derived from microPET images are 2- to 3-times lower than the values obtained from the biodistribution studies. While the uptake data derived from microPET images is lower than that obtained from biodistribution studies, the tumor-to-tissue ratios and the melanoma to non-melanoma tumor uptake ratios were comparable between the microPET images and biodistribution studies. One of the likely explanations for this discrepancy could be due to a mismatch of radioactivity level calibration between the gamma counter and the scanner.

**Fig. 9:**
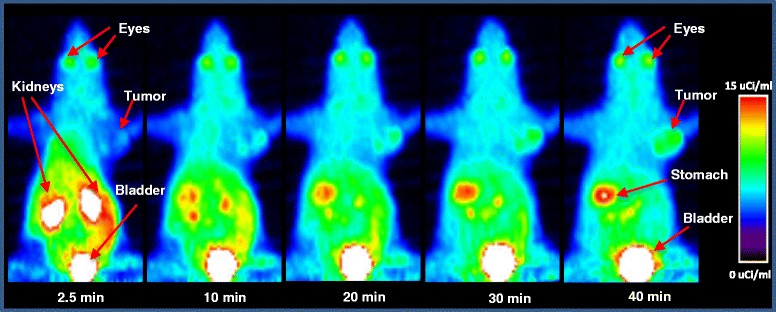
Maximum Intensity projection (MIP) images from MicroPET scans of a mouse bearing B16F1 melanoma tumors xenograft that was injected with ~284 μCi of 4-[^18^F]FEBZA. These MIP images show radioactivity accumulation at 2.5, 10, 20, 30, and 40 min post-injection. Uptake in eyes was quite prominent as early as 2.5 min post-injection. The kidneys show an intense accumulation of radioactivity within 2.5 min post-injection followed by a rapid clearance thereafter. The tumor is clearly visible by 10 min post-injection and the uptake intensity in tumor continued to increase with time

The radioactivity levels were measured from microPET images by drawing ROIs on major organs visible on the scans. The standardized uptake values (SUVs) derived from these microPET images for the B16F1 and HT-29 tumors were 1.53 ± 1.01 and 0.25 ± 0.04, respectively. The MIP image from microPET study for the mouse bearing B16F1 tumor at various time points is shown in Fig. 9.

On these reconstructed images, the B16F1 tumor is clearly visible as early as 10 min post-injection and the uptake intensity continued to increase with time. In addition, a characteristic uptake in the eyes is also distinctly visible in images at all time points.

In summary, 4-[^18^F]FEBZA shows a selective and rapid uptake by melanoma cells in vitro*.* Preferential uptake in melanoma tumor xenograft along with a favorable whole body distribution pattern in vivo suggests a significant potential of this radiotracer in delineating and localizing melanoma.

## Conclusions

Herein, we have developed a rapid and reliable one-step synthesis of 4-[^18^F]FEBZA that provides the desired product in high radiochemical yields and high specific activity. The in vitro studies show a high binding affinity of this compound to melanoma cells. The biodistribution and microPET imaging studies show a rapid and preferential accumulation of 4-[^18^F]FEBZA in melanoma tumors. Results from these preclinical studies suggests that 4-[^18^F]FEBZA is a promising PET imaging probe to target melanoma.
